# Is it time for a new paradigm for systemic cancer treatment? Lessons from a century of cancer chemotherapy

**DOI:** 10.3389/fphar.2013.00068

**Published:** 2013-06-25

**Authors:** Sarah Crawford

**Affiliations:** Cancer Biology Research Laboratory, Southern Connecticut State UniversityNew Haven, CT, USA

**Keywords:** anti-inflammatory, anti-oxidant, tumor microenvironment, chemotherapy, adjuvant, drug resistance, neoplasm

## Abstract

U.S. SEER (Surveillance Epidemiology and End Results) data for age-adjusted mortality rates for all cancers combined for all races show only a modest overall 13% decline over the past 35 years. Moreover, the greatest contributor to cancer mortality is treatment-resistant metastatic disease. The accepted therapeutic paradigm for the past half-century for the treatment of advanced cancers has involved the use of systemic chemotherapy drugs cytotoxic for cycling cells (both normal and malignant) during DNA synthesis and/or mitosis. The failure of this therapeutic modality to achieve high-level, consistent rates of disease-free survival for some of the most common cancers, including tumors of the lung, colon breast, brain, melanoma, and others is the focus of this paper. A retrospective assessment of critical milestones in cancer chemotherapy indicates that most successful therapeutic regimens use cytotoxic cell cycle inhibitors in combined, maximum tolerated, dose-dense acute treatment regimens originally developed to treat acute lymphoblastic leukemia and some lymphomas. Early clinical successes in this area led to their wholesale application to the treatment of solid tumor malignancies that, unfortunately, has not produced consistent, long-term high cure rates for many common cancers. Important differences in therapeutic sensitivity of leukemias/lymphomas versus solid tumors can be explained by key biological differences that define the treatment-resistant solid tumor phenotype. A review of these clinical outcome data in the context of recent developments in our understanding of drug resistance mechanisms characteristic of solid tumors suggests the need for a new paradigm for the treatment of chemotherapy-resistant cancers. In contrast to reductionist approaches, the systemic approach targets both microenvironmental and systemic factors that drive and sustain tumor progression. These systemic factors include dysregulated inflammatory and oxidation pathways shown to be directly implicated in the development and maintenance of the cancer phenotype. The paradigm stresses the importance of a combined preventive/therapeutic approach involving adjuvant chemotherapies that incorporate anti-inflammatory and anti-oxidant therapeutics.

## INTRODUCTION

A new paradigm to guide cancer treatment research may be needed in this 21st century, one that builds upon the previous century of research and discovery on the nature of this very complex and still mysterious disease. An exploration of the highlights and difficulties encountered in the long quest to understand the systemic treatment of cancer may provide a necessary perspective so that we can move forward to realize the long anticipated goal of finding a meaningful and permanent solution to the cancer problem. As the so-called “War on Cancer” rapidly approaches the half-century mark, there has been a lot of discussion about its successes and failures. It is not the purpose of this discussion to elaborate on the political or economic aspects of cancer research, but rather to approach the issue from the standpoint of a very basic scientific inquiry of what we have learned from a century of cancer research with respect to future potential translational and clinical applications.

Cancer chemotherapy emerged as a means for treating systemic disease in the 1960s. Prior to this time, the primary treatment for cancer involved surgery and radiation. Neither therapeutic modality was designed to treat the problem of systemic diseases due to metastasis. This limitation ultimately became the rationale for a new therapeutic approach to deal with the systemic nature of this disease, eventually involving the use of cytotoxic drugs. Before the advent of systemic treatment, the long-term remission rates for cancers across-the-board could not be pushed beyond approximately 35%, and this difficulty, of course, generated much interest in developing systemic approaches that would produce greater cure rates.

The focus of this paper is specifically the biological aspects of cancer therapeutics that comprise the core components of therapeutic responses -both positive and negative- to conventional cytotoxic chemotherapy drugs. This involves an assessment of current chemotherapy modalities and how these therapy approaches may need to be modified in an attempt to develop novel therapeutics for the treatment of refractory cancers.

## CANCER THERAPEUTICS 2013: HOW ARE WE DOING?

**FIGURE 1:**
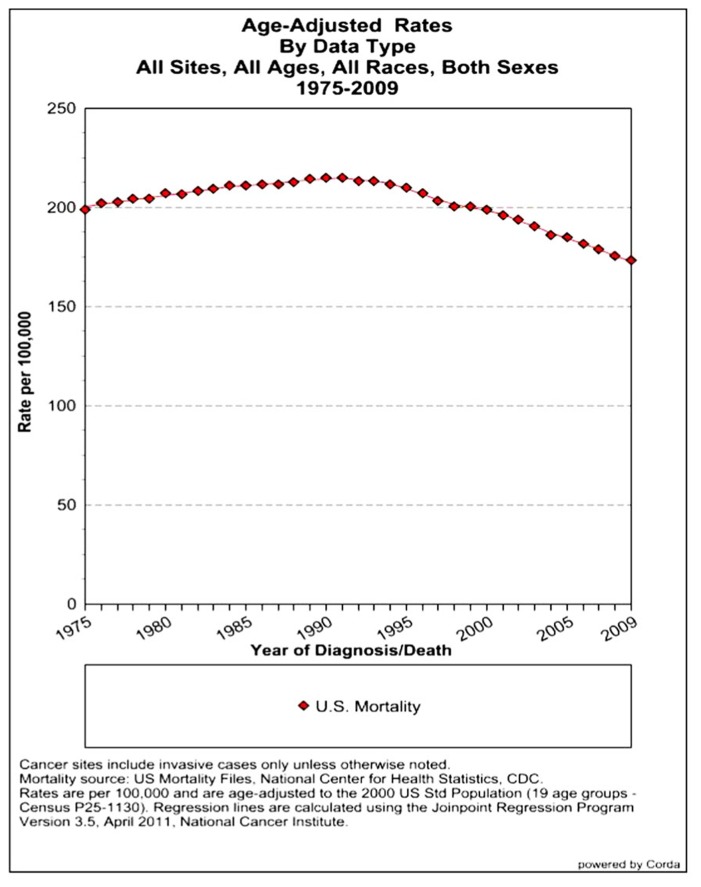
**Age-adjusted mortality rates for all cancers combined, US SEER data (Howlader et al., 2011)**.

**FIGURE 2:**
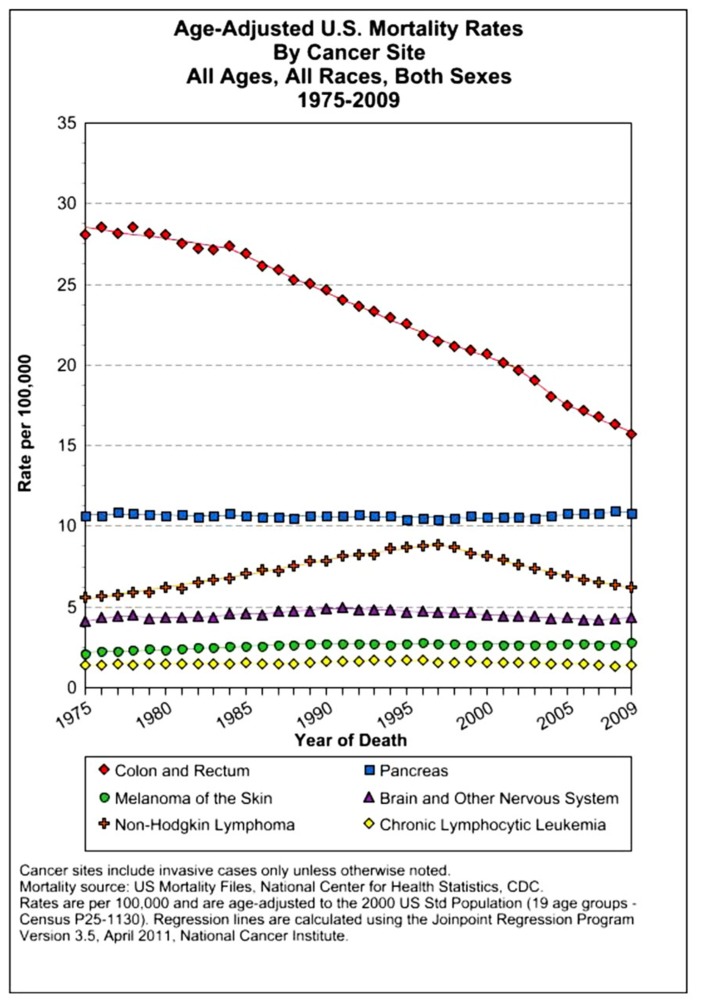
**Age-adjusted mortality rates for specific cancer types in males and females, all races combined, US SEER data (Howlader et al., 2011)**.

**FIGURE 3:**
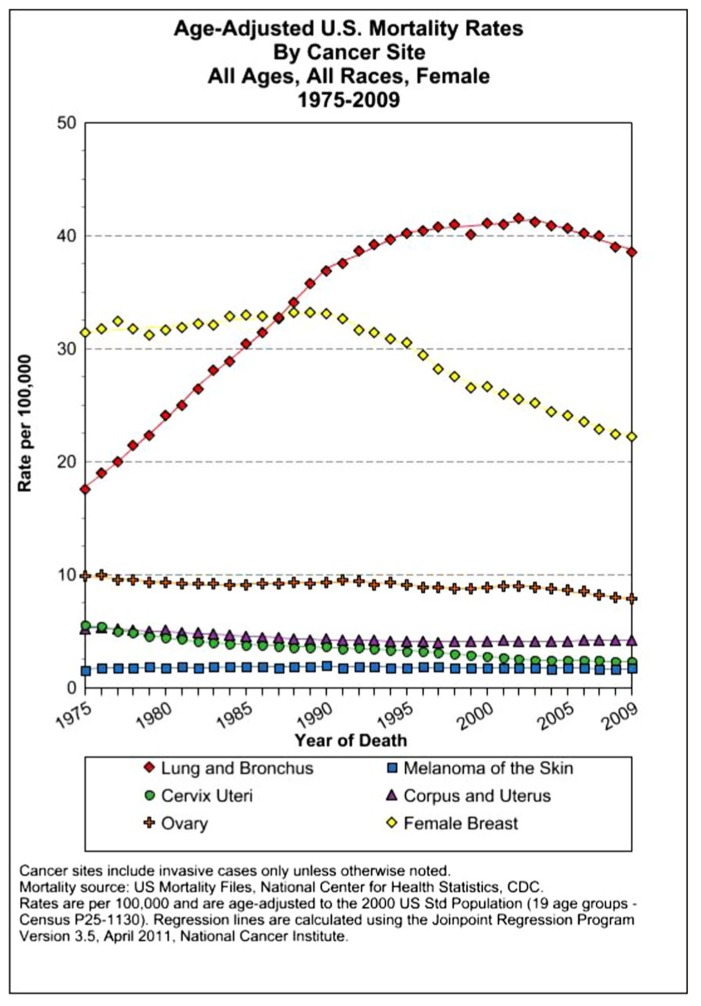
**Age-adjusted mortality rates for specific cancer types in females, all races combined, US SEER data (Howlader et al., 2011)**.

**FIGURE 4:**
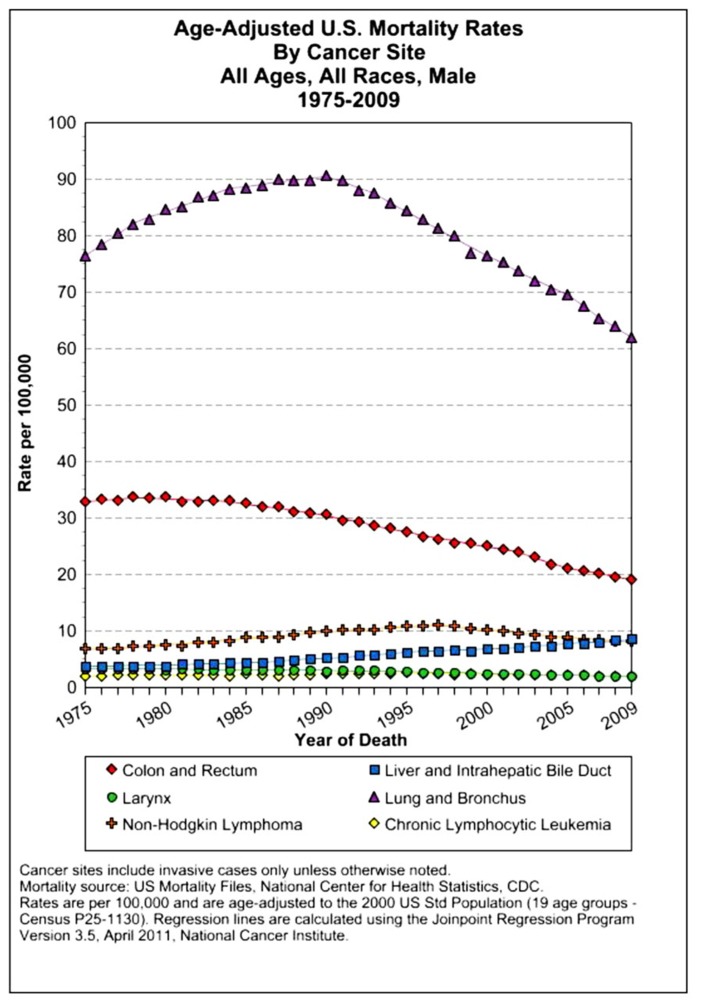
**Age-adjusted mortality rates for specific cancer types in males, all races combined, US SEER data (Howlader et al., 2011)**.

The assessment of cancer statistics: incidence, mortality, and therapeutic efficacy can be a fairly difficult and detailed exercise as one attempts to elucidate these important quantitative parameters of the collection of diseases we call cancer. This quest has perhaps been made even more complicated by the various terminologies that have evolved in evaluations used to assess clinical data, including disease-free survival, time to disease recurrence, overall response rates, and partial/complete responses, to name a few. Despite these complexities, any reading of current cancer statistics suggests that, although there has been significant progress over the past half-century in understanding the basic biology of cancer, the genetics of cancer, and the development of better diagnostic and therapeutic approaches, a consensus of researchers, both basic and clinical, would most likely agree that, in the year 2013, we are not where we would like to be in terms of developing a rational and broadly applicable means of treating many of the most common types of cancer that respond poorly and/or inconsistently to current standard of care treatment approaches. It is beyond the scope of this discussion to present a detailed review of the current statistics; suffice it to say that for many of the most common cancers, including lung cancer, breast cancer, colon cancer, and some of the less common, but, nonetheless, very serious cancers, such as pancreatic cancer and brain cancer, the statistics on successful therapeutic responses have not met with the expectations engendered by the enormous progress in cancer molecular genetics that was made in the latter part of the 20th century (see **Figures 1–4**). **Figure 1** shows the age-adjusted mortality rates for all cancers combined, all ages, all races, and both sexes in the U.S. between 1975 and 2009. The SEER (Surveillance Epidemiology and End Results) data show that the approximate percent decline in mortality rates from all cancers combined has decreased by approximately 13% over the past 40 years (Howlader et al., 2011). **Figure 2** shows the age-adjusted mortality rates for some of the most common cancer sites for all races all genders from 1975 to 2009. **Figure 3** shows mortality rates in women all races for common cancers such as breast and ovarian. **Figure 4** shows age-adjusted mortality rates for common cancers in men all races for this time period.

It is not the purpose of this discussion to elaborate in depth on the specific statistics associated with any particular type of cancer; rather, the focus is to explore in a more general sense what the successes and difficulties of cancer chemotherapy have been over the past 50 years with an eye toward assessing what needs to change in order to make the most of the tremendous scientific resources afforded by the molecular biology advances of the last half-century. There is no doubt that more progress is needed as patient responses to chemotherapy are generally variable even within a single type of cancer and that the term “cure” is one that is only very carefully applied as we look at the potential successes and efficacies of various therapeutic approaches.

## CANCER CHEMOTHERAPY: WHAT DOES HISTORY TELL US?

The treatment of systemic cancers using chemotherapy drugs as a major component of cancer therapeutics began with an early observation made by Beatson (1896) on the effect of estrogens on breast cancer that was noted many years later by Charles Huggins, who was the first to treat men with prostate cancer by castration to block hormone-mediated effects on prostate cancer cells, an approach that resulted in improved patient outcome (Huggins and Hodges, 1941). The historical origins of cytotoxic chemotherapy as a form of systemic cancer treatment began with observations of the physiological effects of agents of chemical warfare such as mustard gas used in World Wars I and II (Gilman, 1946). Clinical studies of soldiers exposed to these agents, followed up by laboratory research in rabbits showed that, among other noxious effects, this type of chemical exposure resulted in the suppression of bone marrow cell proliferation, suggesting a potential clinical application to the treatment of leukemias and lymphomas.

The first human experiments on the use of nitrogen mustard as a cancer chemotherapeutic agent were reported in Chicago in 1943 (Gilman, 1963) when a patient with non-Hodgkin’s lymphoma (NHL) was treated with nitrogen mustard, which produced a dramatic alleviation of disease symptoms. This success ultimately led to the development of other cytotoxic drugs based on the use of nitrogen mustard; these included alkylating compounds such as chlorambucil and cyclophosphamide. These early findings led to the notion of a potential medical use for these agents in the treatment of bone marrow-based blood disorders such as leukemia. The earliest clinical trials were begun in 1946, which represents the beginnings of modern cancer chemotherapy. The first patients were treated with nitrogen mustard gas derivatives, such as myloran and chlorambucil, that produced transient treatment responses that, unfortunately, were followed by inevitable relapses (see below; Goodman et al., 1946).

Some of the most common chemotherapy drugs used today were originally developed as antibiotics intended for use in the treatment of infectious disease. However, some of these antibiotics were found to be too toxic because of their effects on the bone marrow and intestinal lining and were later re-purposed as anti-cancer drugs. For example, para-amino benzoic acid (PABA) derivatives used in cancer chemotherapy were originally developed as anti-microbial sulfa drugs to treat infectious diseases such as streptococcal infections (Pinkel, 1959). This concept that anti-microbial antibiotics could be used also or alternatively as anti-cancer drugs led to the development and the synthesis of the anti-folate antagonists aminopterin and amethopterin (methotrexate; Farber, 1949; Law et al., 1949). These were the first drugs to induce temporary remissions of childhood acute lymphoblastic leukemia (ALL) as early as 1948 (Farber et al., 1948) and also produced the first cures of gestational choriocarcinoma a few years later (Li et al., 1958; Hertz et al., 1963).

**FIGURE 5:**
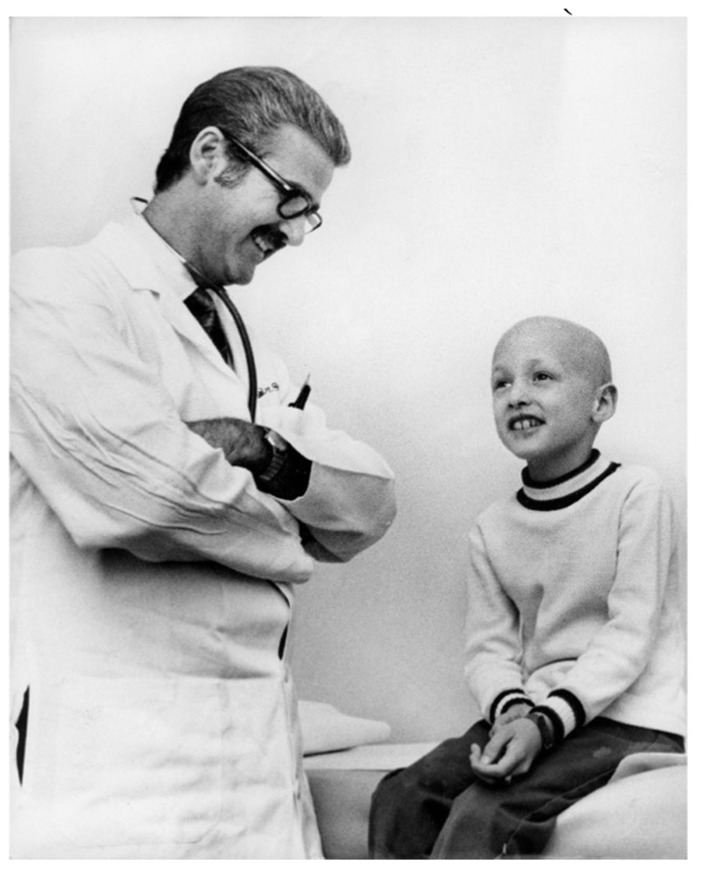
**Development of a successful combined chemotherapy approach for childhood acute lymphoblastic leukemia (ALL) that ultimately became a therapeutic model for the treatment of systemic cancers of many types**. In 2012 the cure rate for childhood ALL is 90%. (Photo: Archive St. Jude’s Hospital). “November 12, 1970 – Dr. Rhomes J. A. Aur, of St. Jude Children’s Research Hospital, with Steven Ray of Jackson, Miss. who has been receiving treatment for leukemia for 2 years. Announcement of a 17% cure rate in leukemia was made at the hospital today.”

These early successes with childhood ALL and choriocarcinoma became the model for the further development of cytotoxic chemotherapy drugs for the treatment of many types of cancer. The first antibiotic developed to take advantage of its anti-tumor properties, 6-mercaptopurine, was discovered in 1948 and developed into a class of purine and pyrimidine antagonists that ultimately were used in the treatment of childhood ALL in the 1950s and 1960s (Elion et al., 1954; Hitchings and Elion, 1954; Frei et al., 1961; George et al., 1968). The drug 5-fluorouracil was discovered at about the same time and was later found to be active against a number of solid tumor malignancies (Heidelberger et al., 1957; see **Figure 5**). Other anti-cancer agents developed in this trial and error approach include the vinca alkaloids that function as anti-mitotic agents (Johnson et al., 1963).

**FIGURE 6:**
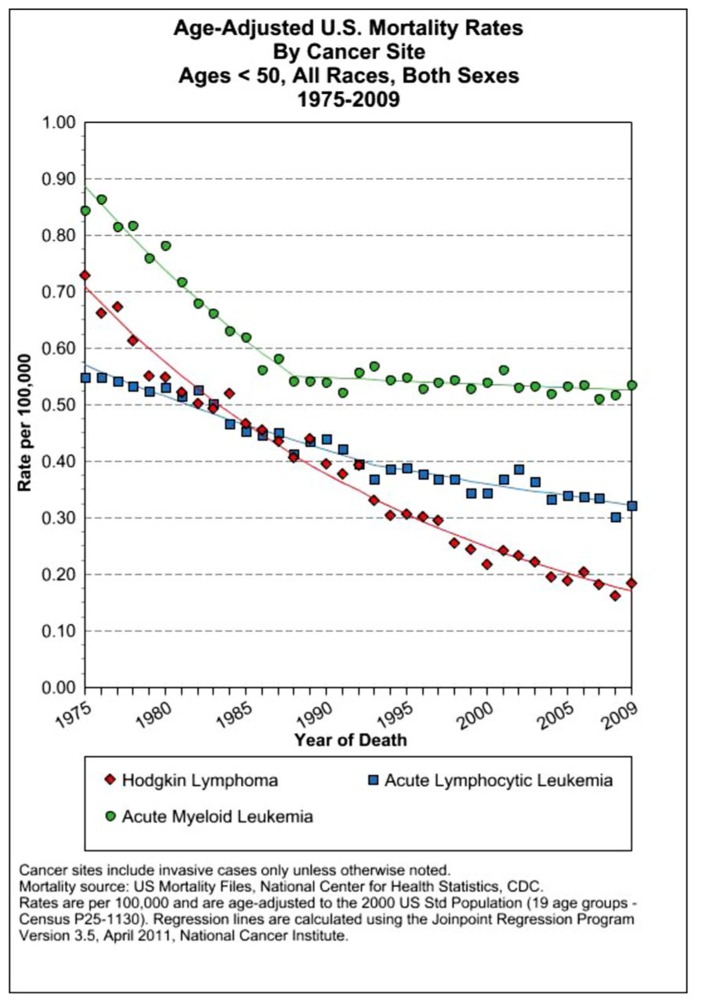
**dUS SEER data on mortality rates of several leukemias and Hodgkin’s lymphoma that have seen a precipitous decline since 1975 resulting from dose-dense combined chemotherapy MTD therapies (Howlader et al., 2011)**.

The development of many important cytotoxic drugs was the result of an effort to identify natural and synthetic compounds with anti-cancer activity via mass screenings for their anti-cancer effects *in vitro* on cultured tumor cell lines. Anti-cancer drugs developed using this trial and error approach include paclitaxel, fludarabine, BCNU, carboplatin, cytosine arabinoside pentastatin, hydroxyurea, topotecan, and mitoxantrone (Marshall, 1964). Most of these are still in widespread use today (see **Figure 6**). Their extraordinary efficacy in the treatment of select cancer types, such as ALL, some types of lymphoma and testicular cancer, is undisputed; nevertheless, the success rate of traditional cytotoxic chemotherapy in producing long-term patient disease-free survival is unpredictable, and in many cancers, unsatisfactory. Based on this clinical record of a half-century of widespread use, it is essential to address the problem of broad-spectrum clinical efficacy of standard chemotherapy in order to maximize its clinical benefit in treatment of cancers most likely to respond to this therapeutic approach.

## PROBLEMS WITH CYTOTOXIC CHEMOTHERAPY: HISTORICAL LESSONS

Very early in the history of cancer chemotherapy, clinical trials producing rapid remissions in patients with ALL and Hodgkin’s disease (HD) were followed by the disappointing recurrence of treatment-resistant disease, soon to be identified as one of the most intractable problems associated with cancer chemotherapy (Hertz et al., 1963; Skipper et al., 1965; Skipper and Perry, 1970). Just as Alexander Fleming noted the growth of penicillin-resistant bacteria in early studies with this antibiotic that presaged the extraordinary clinical problem of antibiotic-resistant “superbugs,” early clinical studies of chemotherapy drugs in cancer patients revealed a similar resistance phenomenon that was to plague the efficacious use of these drugs in the treatment of cancer. Problems associated with their therapeutic efficacy noted from their inception were initial positive treatment responses or remissions that were too often followed by the recurrence of disease that was frequently insensitive to the therapeutic effects of the agent originally used to achieve remission. The term for this phenomenon is “acquired drug resistance.”

Bacterial drug resistance mechanisms were found to result from antibiotic resistance genes that can spread rapidly in populations of bacterial cells and whose presence can be amplified by the selective destruction of bacteria that do not contain these genes, resulting in the “natural selection” of drug-resistant colonies of infectious agents within the body. The same principle has been observed to be responsible for the development of drug-resistant cancer cells; in some cases, drug resistance appears to result from the selection of tumor phenotypes produced by genetic mutations (generally gene amplifications) that confer resistance to the cell killing effects of specific types anti-cancer drugs, such as the amplification of the mdr-1 gene, associated with a multi-drug-resistant phenotype and the dihydrofolate reductase (DHFR) gene, which specifically confers resistance to the folate antagonists (e.g., methotrexate; Schimke, 1988). Thus, one major issue that has emerged from over half a century of use of anti-proliferative chemotherapy drugs is the problem of drug resistance.

This problem has its origins in the genetic instability that is a hallmark of abnormal tumor growth, in which random genetic changes associated with dysregulated proliferative mechanisms are the harbingers of the development of novel genotypes with the capacity to evolve to drug-resistant phenotypes. This is unlike normal dividing cells of the body in which the genetic stability of the cells precludes the development of drug resistance. Malignant cells in general tend to be less stable genetically than normal cells, a phenotype in part associated with dysregulated proliferation (Leach et al., 1993; Kennedy and D’Andrea, 2006). Genetic instability allows for the establishment of drug-resistant clones based upon genetic mechanisms of acquired drug resistance. This phenomenon has provided a rationale for the development of combined, high-dose therapeutic regimens that, in some types of cancer, have been able to prevent the development of acquired resistance (DeVita and Schein, 1973; DeVita et al., 1975; Leach et al., 1993; Rajagopalan and Lengauer, 2004; Kennedy and D’Andrea, 2006).

## THE WAR AGAINST DRUG RESISTANCE LEADS TO NEW CHEMOTHERAPY CLINICAL REGIMENS

In order to bypass this serious clinical issue of drug resistance due to genetic instability, the concept of “combined chemotherapy” evolved, based upon the notion that a combination of drugs with non-overlapping mechanisms of action could prevent or delay the emergence of drug-resistant tumor cells and result in greater overall sensitivity to the cell killing effects of these drugs. The need to prevent drug resistance also led to the development of the concepts of dose intensity and high-dose chemotherapy. Dose intensity refers to a cumulative dose within a specified amount of time involving the maximum tolerated dose (MTD). The concept of dose intensity was based on the observation that patients with HD who received lower dose chemotherapy showed lower cure rates (DeVita and Schein, 1973). Additional, similar observations in patients with breast cancer and colon cancer ultimately led to the application of potentially lethal doses of chemotherapy as a general therapeutic approach to systemic cancers in the hopes of achieving better therapeutic responses (Sparano et al., 2008; Bookman, 2009; Katsumata et al., 2009).

The rationale for these therapeutic approaches was an effort to achieve a maximal and rapid cell killing effect to overwhelm the drug-resistant potential of genetically unstable tumors. To achieve this therapeutic goal, these drugs were delivered at MTDs. This latter approach involves the concept that the higher the dose the greater the therapeutic efficacy and the lower the probability that drug-resistant mechanisms will have the opportunity to develop. This concept led to therapeutic regimens of dose intensity and high-dose chemotherapy in the hope of achieving higher cure rates in advanced cancers of many types (DeVita et al., 1975). With the publication of clinical trial results using combined and high-dose therapeutic regimens, it became clear that combined high-dose cytotoxic chemotherapy treatment could cure cancer (Li et al., 1969; Bookman, 2009; Bonilla et al., 2010).

After a further 25 years of trial and error, it was possible for oncologists to optimize the use of these chemical agents in order to induce long periods of disease-free survival for some types of cancer. High-dose regimens and combined treatment protocols were developed initially for the treatment of childhood leukemia (ALL). The most effective was the VAMP protocol: vincristine, amethopterin, 6-mercaptopurine and prednisone. This therapeutic “cocktail” was administered intermittently to patients to allow for bone marrow recovery. This treatment regimen led to an increase in the rate of remission to 60% by the end of the 1960s (Freireich et al., 1964; Zuelzer, 1964; Frei et al., 1965; Burchenal, 1966; Pinkel et al., 1971).

Another cancer to respond to this therapeutic approach was advanced HD. In the 1960s advanced HD was usually fatal. Remissions were attainable in only 25% of patients; however, disease recurrence was the inevitable result using single agent protocols. The development of the MOMP protocol combining nitrogen mustard with vincristine, methotrexate, and prednisone (which became the MOPP program which omitted methotrexate and replaced it with procarbazine) generated complete remissions at a rate of over 80% with 60% of patients avoiding any relapse of this disease. These clinical results were published in 1965–1967 (DeVita et al., 1965, 1970, 1972; DeVita and Serpick, 1967; Moxley et al., 1967). Beginning in 1975, patients with diffuse large B cell lymphoma showed positive results using a similar protocol that substituted cyclophosphamide for nitrogen mustard. By 1984, mortality from childhood leukemia and HD had fallen by 65% (Papac, 2001). To optimize the therapeutic window, intensive intermittent treatment cycles were given over a series of days to allow recovery of normal proliferating bone marrow cells. These therapeutic regimens led to one of the great success stories of chemotherapy, resulting in dramatic decreases in mortality from the most common form of childhood leukemia (ALL) and lymphoma (HD; see **Figure 6**).

With respect to solid tumor malignancies, it was clear that most patients with local regional disease showing no sign of systemic spread would, nevertheless, inevitably relapse using approaches (surgery and radiation) that target only the site of the original tumor (Greenspan et al., 1963; Canellos et al., 1974a,b). This was clear both in the case of breast cancers as well as in colon and other cancers. Despite the fact that a significant fraction of patients with local regional disease do not relapse, the rationale for the use of adjuvant chemotherapy as an adjunct to local regional treatments was the evidence that the percentage of patients relapsing would be high in the absence of adjuvant systemic therapy (Budman et al., 1998; Fisher et al., 1999).

Clinical support for the rationale that combined chemotherapy could be useful in the treatment of solid tumors resulted from clinical results obtained in the 1960s and 1970s indicating that combined chemotherapy could cure some types of advanced cancer (DeVita and Schein, 1973). Theoretical support for adjuvant chemotherapy was provided by Skipper’s cell kill hypothesis suggesting that, if tumors were treated at the level of micro-metastases rather than as larger volume tumors, it would be more likely that treatment would be effective (Skipper and Perry, 1970; Schabel, 1975). These early successes in the 1960s and 1970s led to the widespread application of these approaches to many different types of human cancers, including many types of solid tumor malignancies.

One of the first adjuvant chemotherapy approaches in the treatment of solid tumors combined cyclophosphamide, methotrexate, and 5-fluorouracil as an adjuvant chemotherapy for patients with metastatic cancer [the CMF (Cyclophosphamide Methotrexate Fluorouracil) regimen]. The overall response rate was over 50%; about 20% of patients showed complete remissions. The CMF study was published in 1976 with positive results (Canellos et al., 1974a; Bonadonna et al., 1976).

Testicular cancer remissions went from 10 to 60% starting in 1978 using a combination of cisplatin, elastin, and bleomycin (Li et al., 1969; Einhorn and Donohue, 1977, 1979). A notable successful application of this approach is the use of the PVB protocol (platinum, vinblastine, bleomycin) combined chemotherapy for testicular cancer (Einhorn, 1981). A large number of additional studies on adjuvant therapy for breast cancer as well as other cancers such as colon cancer produced positive results that contributed to a modest decline in U.S. mortality from breast cancer, ovarian cancer, and others (Sparano et al., 2008; Bookman, 2009; Katsumata et al., 2009; Bonilla et al., 2010).

For the past several decades, the prevailing therapeutic paradigm for the treatment of both disseminated leukemias/lymphomas as well as almost all solid tumor malignancies has involved the use of dose-dense, combined MTD cytotoxic chemotherapy to mitigate the problem of drug resistance. Despite these broad-spectrum applications to almost all currently used chemotherapy regimens, in only a few types of cancer do we see curative responses such as have been consistently observed in the treatment of childhood ALL and HD (see **Figures 2–4**).The results of over a half-century of clinical trials have shown that the therapeutic approach of combined/dose-dense chemotherapy has not been entirely successful in achieving its primary purpose, which is the induction of long-term disease-free survival in the majority of patients with systemic disease (Kaufmann et al., 2011). Thus, the rational use of these conventional chemotherapy treatment protocols is challenged by the failure to observe consistent results associated with long-term remissions of many common cancers. Moreover, these clinical data indicate that the problem of chemotherapy resistance is greater than that defined by acquired drug resistance mechanisms which would account for the inability of dose-dense, combined MTD approaches to provide greater efficacy in combating this problem. Research in solid tumor biology suggests a more fundamental cause for chemotherapy resistance involving biological mechanisms intrinsic to the cancer phenotype that are directly responsible for the limited efficacy of these current chemotherapy approaches to the treatment of solid tumor malignancies. The primary reason for treatment failure can be found in the biological properties of the malignant system, termed “intrinsic resistance.”

## LEUKEMIAS, SOLID TUMORS, MAXIMUM TOLERATED GROWTH, AND THE “CELL KILL” PARADIGM

The mechanism of both phase specific and non-phase specific chemotherapy drugs (which encompasses the vast majority of cytotoxic chemotherapy drugs in current clinical use) is to target dividing cells (Kinzler and Vogelstein, 1998). Most of the drugs currently used in cancer chemotherapy were developed between the years of 1953 and 1983. Today, there are more than 75 Food and Drug Administration (FDA) approved anti-cancer drugs. Almost all of these are cytotoxic drugs that share a similar mechanism of action to the extent that their toxic effects are specifically targeted to dividing cells, due to interference with DNA metabolism or mitosis (see **Table 1**).

**Table 1:** A list of commonly used cytotoxic chemotherapy drugs that block DNA metabolism or cell division (mitosis) along with their most common clinical indications and side effects (NCCN.com, the National Comprehensive Cancer Network)^®^.

Chemotherapy drug	Possible side effects (Not all side effects are listed. Some of those listed may be short-term side effects; others are long-term side effects)
Carboplatin (paraplatin) Usually given intravenously (IV) – used for cancers of the ovary, head and neck, and lung	Decrease in blood cell counts, hair loss (reversible), confusion, nausea, vomiting, and/or diarrhea (usually a short-term side effect occurring the first 24–72 h following treatment)
Cisplatin (platinol, platinol-AQ) Usually given intravenously (IV) – used for cancers of the bladder, ovary, and testicles	Decrease in blood cell counts, allergic reaction, including a rash and/or labored breathing, nausea and vomiting that usually occurs for 24 h or longer, ringing in ears and hearing loss, fluctuations in blood electrolytes, kidney damage
Cyclophosphamide (Cytoxan, Neosar) Can be given intravenously (IV) or orally – used for lymphoma, breast cancer, and ovarian carcinoma	Decrease in blood cell counts, nausea, vomiting, abdominal pain, decreased appetite, hair loss (reversible), bladder damage, fertility impairment, lung or heart damage (with high doses), secondary malignancies (rare)
Doxorubicin (adriamycin) Given intravenously (IV) – used for breast cancer, lymphoma, and multiple myeloma	Decrease in blood cell counts, mouth ulcers, hair loss (reversible), nausea and vomiting, heart damage
Etoposide (VePesid) Can be given intravenously (IV) or orally – used for cancers of the lung, testicles, leukemia, and lymphoma	Decrease in blood cell counts, hair loss (reversible), nausea and vomiting, allergic reaction, mouth ulcers, low blood pressure (during administration), decreased appetite, diarrhea and abdominal pain, bronchospasm, flu-like symptoms
Fluorouracil (5-FU) Given intravenously (IV) – used for cancers of the colon, breast, stomach, and head and neck	Decrease in blood cell counts, diarrhea, mouth ulcers, photosensitivity, dry skin
Gemcitabine (Gemzar) Given intravenously (IV) – used for cancers of the pancreas, breast, ovary, and lung	Decrease in blood cell counts, nausea and vomiting, fever and flu-like symptoms, rash
irinotecan (Camptosar) Given intravenously (IV) – used for cancers of the colon and rectum	Decrease in blood cell counts, diarrhea, hair loss (reversible)
Methotrexate (Folex, Mexate, Amethopterin) May be given intravenously (IV), intrathecally (into the spinal column), or orally – used for cancers of the breast, lung, blood, bone, and lymph system	Decrease in blood cell counts, nausea and vomiting, mouth ulcers, skin rashes and photosensitivity, dizziness, headache, or drowsiness, kidney damage (with a high-dose therapy), liver damage, hair loss (reversible), seizures
Paclitaxel (Taxol) Given intravenously (IV) – used with cancers of the breast, ovary, and lung	Decrease in blood cell counts, allergic reaction, nausea and vomiting, loss of appetite, change in taste, thin or brittle hair, joint pain (short-term), numbness or tingling in the fingers or toes
Topotecan (Hycamtin) Given intravenously (IV) – used for cancers of the ovary and lung	Decrease in blood cell counts, diarrhea, hair loss (reversible), nausea and vomiting
Vincristine (Oncovin, Vincasar PFS) Usually given intravenously (IV) – used for leukemia and lymphoma	Numbness or tingling in the fingers or toes, weakness, loss of reflexes, jaw pain, hair loss (reversible), constipation or abdominal cramping
Vinblastine (Velban) Given intravenously (IV) – used for lymphoma and cancers of the testis and head and neck	Decrease in blood cell counts, hair loss (reversible), constipation or abdominal cramping, jaw pain, numbness or tingling in the fingers or toes

*Stanford Medicine – School of Medicine – Stanford Cancer Center – Understanding Cancer*.

*^©^ 2013 Stanford Medicine Terms of UsePowered by IRT*.

Their mechanisms of action, therefore, do not distinguish between normal and cancerous proliferating cells, a primary cause of side effects such as bone marrow suppression and dose-limiting toxicities. The net result is a fairly narrow therapeutic window between anti-tumor effect and MTD. These chemotherapy drugs are, therefore, more accurately classified as anti-proliferative, rather than anti-cancer agents. The therapeutic window associated with differential responses between tumor cells and normal dividing cells is based on differences in proliferation rates and recovery parameters that may favor the tumor target, particularly in fast growing malignancies. These differences in normal versus tumor cell sensitivity to agents that block cell division, however, are insufficient to prevent cytotoxic side effects in normal bone marrow and normal dividing epithelial cells. These overlapping sensitivities ultimately limit the MTD, which may be insufficient to destroy all tumor cells without causing irreparable damage to normal host tissue. Moreover, the ablation of the bone marrow has a significant effect on the immune system, at least temporarily decreasing its capacity to defend against the malignant growth within the body.

The vast majority of chemotherapy drugs used in “standard of care” oncology practice for the treatment of advanced cancers have a mechanism of action defined by the cell kill paradigm. The cell kill hypothesis originated with Skipper based on studies of L1210 mouse (Skipper et al., 1965; Skipper and Perry, 1970). The cell kill hypothesis states that a specific dose of drug kills a constant fraction of cells rather than a specific number. Its incremental success, therefore, depends on the number of cells at the start of the treatment. The cell kill hypothesis ultimately became the theoretical rationale for the implementation of high-dose chemotherapy treatment regimens (Norton, 1985, 1988). This conceptual approach was further enabled by the development of autologous peripheral stem cell procedures in the 1980s and 1990s that involve dose-dense chemotherapy in conjunction with ablation of the bone marrow and immune system followed by autologous peripheral stem cell rescue. This approach represents a full-scale application of the cell kill paradigm that has been widely used to treat many types of advanced systemic cancers. Unfortunately, application of this therapeutic approach to solid tumor malignancies has not generally produced similar success rates. The primary reason is that the cell kill paradigm cannot be applied to the kinetics of solid tumor growth.

## DIGGING DEEPER: GROWTH PARAMETERS OF SOLID TUMORS CAUSE CHEMOTHERAPY RESISTANCE

The narrow therapeutic window limits the MTD of cytotoxic chemotherapy drugs that target dividing cells. A very important side effect of this limitation is that the rate of cell division in the interior regions of solid tumors is frequently extremely low, lower than normal dividing cells, thereby bypassing the cytotoxic effects of these drugs even at their MTDs. What accounts for the low rate of cell division in the solid tumor? The answer can be found in the abnormal microenvironment that develops as the solid tumor mass enlarges.

The clinical use of most conventional chemotherapy drugs involves the basic assumption that the cancer phenotype is characterized by continuous dysregulated cell proliferation. However, research on tumor cell biology suggests that many solid tumors of diverse tissue types are heterogeneous collections of cells that are not consistently or uniformly proliferating at any given time (Norton et al., 1976; Norton, 1985, 1988). Fundamental difference between the growth properties of leukemias/lymphomas and solid tumors may explain key differences in chemotherapy sensitivities.

**FIGURE 7:**
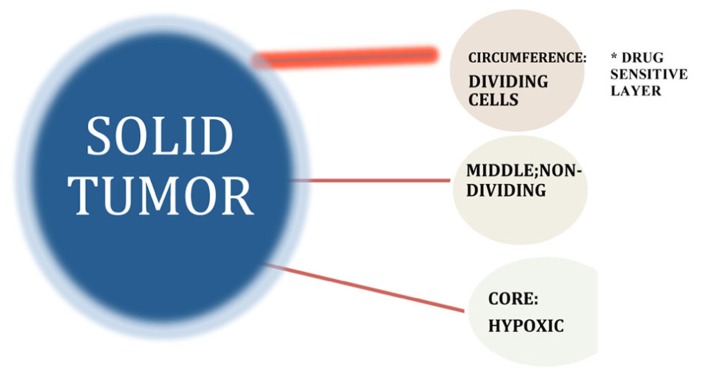
**Growth fraction model suggests that tumors consist of pools of both proliferating and non-proliferating cells, with only the former category possessing the intrinsic biological capacity to respond to drugs that specifically target dividing cells**. Differential localization of cells within a solid tumor give rise to marked gradients in the rate of cell proliferation as a result of decreasing diffusion rates for oxygen, nutrients, and growth factors-associated abnormal vascularization of the solid tumor interior. Intrinsic chemotherapy resistance results when non-dividing cancer cells do not respond to S- and M-phase chemotherapy drugs that block cell proliferation by virtue of the fact that they are not in either the S- or M-phase of the cell cycle at the time of treatment (Komarova and Wodarz, 2005).

The biological properties of solid tumors generally differ significantly from those of the disseminated cancers and profoundly affect critical therapeutic parameters (Nederman and Carlsson, 1984; Tunggal et al., 1999; Tannock et al., 2002). One of the most important differences, as it relates to sensitivity to cell cycle inhibitors, involves the heterogeneous proliferation rates that characterize solid tumors (Lupi et al., 2004). Unlike leukemic cells that are released from the bone marrow at a premature stage of differentiation and disseminated in the circulation, in solid tumors transformed cells originate and proliferate as a solid mass at a specific site of origin (at least, prior to metastasis) generating abnormal, multi-dimensional structures that produce a distinctive microenvironment associated with unique biophysical, biochemical, and physiological properties (Teicher et al., 1990; Kerbel et al., 1996; Vaupel, 2004; Kozusko et al., 2007). Differential localization of cells within a solid tumor give rise to marked gradients in the rate of cell proliferation as a result of decreasing diffusion rates for oxygen, nutrients, and growth factors associated with absent or abnormal vascularization of the solid tumor interior (Hirst and Denekamp, 1979; Vaupel et al., 1989; Kinzler and Vogelstein, 1998; Ljungkvist et al., 2002; Roskelley and Bissell, 2002; see **Figure 7**). This results in regions of hypoxia and acidity that can also influence the sensitivity of the tumor cells to drug treatment (Durand, 1986; Olive and Durand, 1994; Lankelma et al., 1999).

Tumor geometry and the altered microenvironment that result from solid tumor growth may thus play critical roles in the resistance of solid tumors to chemotherapy and must be included in any relevant discussion of potential strategies to improve the effectiveness of drug treatment of solid tumor malignancies. Unfortunately, application of this therapeutic approach to solid tumor malignancies has not generally produced similar success rates. The primary reason is that the cell kill paradigm cannot be applied to the kinetics of solid tumor growth. The cell kill paradigm as reflected in the clinical use of most conventional chemotherapy drugs involves the basic assumption that the cancer phenotype is characterized by continuous dysregulated cell proliferation. However, research on tumor cell biology suggests that many solid tumors of diverse tissue types are heterogeneous collections of cells that are not consistently or uniformly proliferating at any given time (Norton et al., 1976; Norton, 1985, 1988; Lupi et al., 2004). These biological properties of solid tumors generally differ significantly from those of the disseminated cancers and profoundly affect critical therapeutic parameters (Nederman and Carlsson, 1984; Tunggal et al., 1999; Tannock et al., 2002). Unlike leukemic cells that are released from the bone marrow at a premature stage of differentiation and disseminated in the circulation, in solid tumors transformed cells originate and proliferate as a solid mass at a specific site of origin (at least, prior to metastasis) generating abnormal, multi-dimensional structures that produce a distinctive microenvironment associated with unique biophysical, biochemical, and physiological properties (Teicher et al., 1990; Kerbel et al., 1996; Vaupel, 2004; Kozusko et al., 2007). Differential localization of cells within a solid tumor give rise to marked gradients in the rate of cell proliferation as a result of decreasing diffusion rates for oxygen, nutrients, and growth factors associated with absent or abnormal vascularization of the solid tumor interior (Hirst and Denekamp, 1979; Vaupel et al., 1989; Kinzler and Vogelstein, 1998; Ljungkvist et al., 2002; Roskelley and Bissell, 2002). This results in regions of hypoxia and acidity that can also influence the sensitivity of the tumor cells to drug treatment (Durand, 1986; Olive and Durand, 1994; Lankelma et al., 1999). Tumor geometry and the altered microenvironment that results from solid tumor growth may thus play critical roles in the resistance of solid tumors to chemotherapy and must be included in any relevant discussion of potential strategies to improve the effectiveness of drug treatment of solid tumor malignancies.

## LEUKEMIAS/LYMOHOMAS VERSUS SOLID TUMORS: DIFFERENT DISEASE REQUIRE DIFFERENT THERAPEUTIC APPROACHES

A critical historical development in cancer treatment approaches involved the wholesale application of chemotherapy drugs used in the successful treatment of some cancers arising in the reticuloendothelial system (i.e., leukemias and lymphomas) to the treatment of solid tumor malignancies (Panetta et al., 2006). These therapeutic protocols were developed initially in the context of treating childhood leukemias and lymphomas; their extended clinical applications to the treatment of solid tumor malignancies were based largely on the therapeutic successes observed, for example, in the treatment of HD and acute lymphoblastic childhood leukemia (ALL). In this context, therapeutic modeling in solid tumors was largely based on the growth parameters defined by the growth of leukemia cells (Kim and Tannock, 2005). However, the vast differences in growth properties that distinguish solid tumor growth from that of the leukemias and some lymphomas made this translational approach inappropriate. Leukemias and solid tumor malignancies are very different biological entities. Although they share similar growth properties in that all types of cancer ultimately suffer from regulatory dysfunction in proliferation that is often associated with blocks to cell differentiation pathways, nevertheless, solid tumor malignancies have a biology that is significantly different from that of the disseminated malignancies. The primary therapeutic target in the leukemias/lymphomas is the abnormal cancer stem cell population of the bone marrow/lymph node. This target is more amenable to treatment with cytotoxic drugs that block cell cycle proliferation than are solid tumors, as the propagation of abnormally dividing cells comprises the primary cancer phenotype. In contrast, solid tumor malignancies arise and accumulate as abnormal masses of growth dysregulated cells enmeshed in the tissue of origin. These tumor masses, as well as their metastatic counterparts, develop a phenotype that is a product not only of genetically induced cell cycle dysregulation, but also as a consequence of the abnormal microenvironment created by the tumor mass (Durand, 1986; Olive and Durand, 1994). The net result is a tumor whose proliferative capacity is generally restricted to its outer margins, thereby seriously limiting the potential efficacy of cancer drugs that target dividing cells. Moreover, the abnormal microenvironment presents a barrier to drug uptake and critical mechanisms of action that depend on reactive oxygen species (ROS) production (Pouyssegur et al., 2006). Most importantly, solid tumor growth generates an abnormal regulatory equilibrium in which tumor survival depends less on the sustained activation of a small subset of dysregulated genes but rather on the epigenetic effects of the tumor microenvironment that ultimately sustain tumor survival and spread (Lankelma et al., 1999; Roskelley and Bissell, 2002). The biological selection parameters that drive this abnormal equilibrium may define limits on the potential efficacy of therapeutics designed to attack solid tumor growth and survival by abrogating the activities of selected dysregulated genes that drive tumor initiation but may exercise only a limited role in sustaining systemic disease progression (Sutherland et al., 1979; West et al., 1980; Wibe, 1980; Coley et al., 1993; Kuh et al., 1999; Au et al., 2002; Grantab et al., 2006; Minchinton and Tannock, 2006; Di Paolo and Bocci, 2007).

## GROWTH FRACTION AND THE SOLID TUMOR PHENOTYPE

Mendelssohn’s concept of defined growth fraction attempts to define the kinetic basis for observed non-exponential growth patterns of many human cancers (Mendelsohn, 1961). This concept postulates that a tumor cell population simply consists of two pools: one that is proliferating and one that is not proliferating. A proposed explanation for why many human tumors do not proliferate exponentially or, therefore, respond to anti-chemotherapy drugs that block cell proliferation by first order kinetics is based this overall difference in cell division rates between normal and tumor cells expressed as the tumor growth fraction. Gompertz (1825) was the first to propose a non-exponential growth pattern to characterize the growth behavior of human cancers (Norton et al., 1976; Norton and Simon, 1977a,b; Norton, 1988; Abbott and Michor, 2006; Kozusko et al., 2007). He argued that tumors essentially grow in a sigmoidal pattern such that the fastest growth rate is observed when tumors are about one third of their maximum size (volume) and that slower growth rates are observed at either end of the growth curve. This theory suggests that small tumors and micro-metastases should be more sensitive to cell division inhibitors used in chemotherapy because their growth rates are higher than very large tumors and, therefore, at a stage more sensitive to the cytotoxic effects of agents that block cell proliferation. This prediction, however, has not been borne out in clinical and pre-clinical observations of small volume tumor/micro-metastases sensitivity parameters to S- and M-phase inhibitors, as they often display a drug-resistant phenotype (Tannock, 1978; Abbott and Michor, 2006). It is generally accepted that these micrometastatic lesions display chemosensitivity patterns resulting from acquired resistance mechanisms observed subsequent to primary chemotherapy-induced remissions.

This issue of tumor cell division rates and its relationship to cancer drugs that specifically target cycling cells with respect to the problem of drug resistance may, therefore, in part be explained by the growth fraction model that suggests that tumors consist of pools of both proliferating and non-proliferating cells, with only the former category possessing the intrinsic biological capacity to respond to drugs that specifically target dividing cells (Kozusko et al., 2007). In this context, intrinsic chemotherapy resistance can be defined as the failure of non-dividing cancer cells to respond to S- and M-phase chemotherapy drugs that block cell proliferation by virtue of the fact that they are not in either the S- or M-phase of the cell cycle at the time of treatment (Komarova and Wodarz, 2005).

The biological parameters responsible for the growth behavior of solid tumors, as defined by the growth fraction, may be explained in part by the observed growth behavior of micro-metastases *in vitro* that follow spheroidal growth parameters of volumetric increases that create a changing environment surrounding the cells of the tumor, depending upon their location within the solid tumor (Sutherland et al., 1979; West et al., 1980; Wibe, 1980; Kuh et al., 1999). At the exterior, there is a shell of proliferating cells up to five to six layers that are directly exposed to physiological levels of growth factors, nutrients, and oxygen. These parameters change as cells of the interior layers of the tumor comprising the so-called “middle layer” experience some degree of deprivation of these growth stimulants based on avascular diffusion rates as these factors move through the tumor layers. The decreased availability of nutrients, growth factors and oxygen block cell proliferation within this middle layer, that nevertheless, remains substantively viable (Sutherland et al., 1979; Coley et al., 1993; Tannock et al., 2002). At the deepest internal layers of the tumor, the innermost core often becomes necrotic due to very low-level exposure to oxygen and nutrients. This model of tumor growth for small (1–3 mm) avascular tumor masses may explain some aspects of drug resistance based on the fact that the use of cell cycle inhibitors would be expected to destroy selectively the outermost proliferating shell of the tumor, but not have a significant effect on the inner non-proliferating cell layers that comprise the bulk of the tumor mass. The biological basis of growth fraction may thus reside in the geometry of spheroidal growth and the local microenvironmental changes generated by the developing tumor mass that may define cell proliferation rates within the tumor and, concomitantly, intrinsic drug resistance to conventional chemotherapy (Au et al., 2002; Grantab et al., 2006).

A second component of solid tumor biology that may seriously diminish the efficacy of traditional cytotoxic chemotherapy drugs is the unique microenvironment that develops during the establishment of solid tumors. In addition to limiting the proliferative capacity of tumor cells in the solid tumor interior, these microenvironmental differences may directly contribute to tumor resistance to the cytotoxic effects of these drugs (Minchinton and Tannock, 2006; Di Paolo and Bocci, 2007). For example, as the tumor interior becomes increasingly hypoxic due to poor and inefficient vascularization, resistance to death pathways that depend on the production of oxygen free radicals may produce a drug-resistant phenotype (Hirst and Denekamp, 1979; Ljungkvist et al., 2002). This may be extremely relevant to the problem of drug resistance as many S-phase inhibitors that damage DNA (and also therapeutic radiation) rely upon free radical production as a primary pathway to induce cell death; therefore, this microenvironmental component of solid tumors may be an important factor limiting the toxic effects of these therapeutic agents (Vaupel et al., 1989; Bussink and van der Kogel, 2003; Koukourakis et al., 2006).

## NEW PARADIGM: INFLAMMATION AND REACTIVE OXYGEN SPECIES UNDER ATTACK

The limited and inconsistent success rate resulting from the current treatment regimens of many cancers needs to be strengthened. How do we go about doing this in a way that will make the most sense, based upon our understanding of cancer biology, the lessons that we may take from more than a half-century of therapy involving the use of traditional chemotherapeutic drugs and the newer, targeted biologic drugs? This new therapeutic paradigm represents a synthesis of what is currently understood about the biological processes that lead to the development of malignancies and the results that have been obtained from therapeutic and preventive studies that have been ongoing for the past half-century, highlighting those approaches which appear to have met with the greatest success in identifying potential reliable approaches that might be exploited for therapeutic purposes over the next decade.

### INFLAMMATION

Researchers have long understood that there is a relationship between inflammation and cancer, though the mechanisms involved were obscure. Rudolf Virchow was one of the first biologists to suggest this association in the 19th century. More recently, inflammatory pathways associated with the development of some of the most common cancers have been elaborated and explained at the molecular level. Major risk factors for the most common cancers include chronic infection, obesity, alcohol, tobacco, radiation, high calorie diets, and environmental pollutants. Moreover, each of these risk factors has been shown to contribute to cancer development vis-a-vis inflammatory processes. Research has shown that long-term inflammation has been linked to most chronic illnesses: cancer, cardiovascular disease, diabetes and obesity (Braun et al., 2009; Khandekar et al., 2011). There is substantial evidence that many cancers, especially solid tumors such as colon cancer and pancreatic cancer, are preceded by inflammation within the organ in which the cancer arises (Rolland et al., 1980; Kune et al., 1988; Coussens and Werb, 2002; Farrow and Evers, 2002; Pai et al., 2002; MacArthur et al., 2004; Nelson et al., 2004; Philip et al., 2004). For example, 15–20% of smokers with chronic obstructive pulmonary disease will develop lung cancer (Turner et al., 2007). Colitis is associated with high risk of colon cancer; infection with H. pylori is linked to stomach cancer (Pai et al., 2002; Peek and Blaser, 2002; Itzkowitz and Yio, 2004; MacArthur et al., 2004). These and other research studies have shown that chronic inflammation precedes the development of many types of cancer, and that inflammatory pathways are constitutively active in most cancers (Rolland et al., 1980).

Most of the risk factors associated with inflammation have been to shown to activate NF-kB (nuclear factor-kappaB) and STAT-3 (signal transducer and activator of transcription 3), major transcription factors that regulate inflammatory pathways (Ivanenkov et al., 2011). NF-kB was discovered in lymphoid cells in 1986 and ultimately found to be a ubiquitous transcription factor found in all cells (Sen and Baltimore, 1986). STAT-3 is a transcriptional regulator found in the cytoplasm of most cells and is activated in response to certain inflammatory stimuli resulting in the production of gene products such as the BCL-XL (B-cell lymphoma-extra large) and other growth factors; most recently, dysregulated function of STAT-3 has been linked to cancer metastasis (Deng et al., 2012). Most agents that promote inflammation activate NF-kB, including endotoxins, carcinogens, radiation, chemotherapy, hyperglycemia, tumor promoters, inflammatory cytokines [e.g., tumor necrosis factor (TNF), interleukin 1 (IL-1)], and growth factors such as epidermal growth factor (EGF). In addition, almost all infectious agents associated with cancer activate NF-kB, including human papilloma virus (HPV), human herpesvirus (HHV), hepatitis B virus (HBV), and hepatitis C virus (HCV; Peek and Blaser, 2002; Itzkowitz and Yio, 2004).

Pro-inflammatory stimuli such as TNF, IL-1 and IL-6, cyclo-oxygenase-2 (COX-2), and lipoxygenase (LOX) all regulate NF-kB and are expressed in bronchitis, colitis, gastritis, and hepatitis (Philip et al., 2004). Moreover, chemotherapy and radiation activate NF-kB, which subsequently contributes to acquired chemo-resistance. Tumor pathways associated with survival, proliferation, invasion, and metastasis are all important activators of NF-kB (MacArthur et al., 2004) in positive feedback loop mechanisms that may drive advanced cancer progression. Based on a large volume of research data, including the aforementioned and other studies, one may conclude that most gene products associated with inflammation can contribute to cancer development as well as progression; survival, proliferation, invasion, angiogenesis, and metastasis, all of which are regulated by NF-kB and STAT-3 (Rolland et al., 1980; Sheng et al., 2001; Pai et al., 2002; Chang et al., 2004).

Much recent attention has focused on the long-term use of anti-inflammatory drugs such as aspirin as cancer preventives. The association between chronic inflammation and inflammatory pathway activation in the genesis of cancer is supported by clinical research studies that provide evidence for the preventive aspects of long-term aspirin use in the development of some common malignancies, including breast, colon cancer, bladder cancer, melanoma, and other cancers (Castelao et al., 2000; Sharpe et al., 2000; Anderson et al., 2002; Thun et al., 2002; Pereg and Lishner, 2005; Ulrich et al., 2006; Annemijn et al., 2012). This preventive effect has even been documented in individuals at high risk for developing colon cancer due to inherited genetic mutations (Ulrich et al., 2006; Burn et al., 2011; Chan and Lippman, 2011).

Additional research studies suggest that suppression of inflammatory pathways may be involved in both the prevention and treatment of cancer (Sharpe et al., 2000; Anderson et al., 2002; Thun et al., 2002; Baek and Eling, 2006; Eling et al., 2006). Suppression of the NF-kB regulator, IKKB (inhibitor of kappaB kinase beta), and STAT-3 have been shown to block tumor proliferation and invasion (Pereg and Lishner, 2005). Difluoromethylornithine and sulindac were shown to reduce the risk of colorectal adenoma recurrence by 70%.

Several recently published studies have provided further evidence for preventive and therapeutic effects of daily aspirin. Longitudinal studies have shown that long-term daily aspirin use for at least 10 years reduces the risk of developing colon cancer and other common cancers. These clinical data indicated that low-dose aspirin use for at least 3 years can reduce the risk of cancer incidence by about 25% and the risk of dying from cancer by about 15%. The statistic increases from 25 to 37% for those who take aspirin for longer than 5 years (Rothwell et al., 2012). The results of these studies showed that aspirin use helped prevent the spread or metastasis of cancer to other organs (Annemijn et al., 2012; Rothwell et al., 2012). These data suggested that long-term daily aspirin use reduced the proportion of cancers that spread systemically by 48%. Moreover, use of anti-inflammatory drugs reduced the risk of being diagnosed with a solid cancer that had already spread by 31%. For patients initially diagnosed with a local cancer, the risk of later metastasis was reduced by 55% by daily high-dose aspirin usage. The study authors suggested that at least part of this preventive effect may be linked to the effects of aspirin on platelets. Moreover, the authors suggested that theirs was the first study to show that ANY drug could reduce metastasis as a specific drug-induced effect.

Additional natural products with demonstrated anti-cancer activity that also block inflammation via NF-kB pathway inhibition include curcumin, resveratrol, ursolic acid, and others (Huang et al., 1994; Jang et al., 1997). For example, in human clinical trials, curcumin was shown to down-regulate NF-kB and STAT-3 and is thought to have potential for the prevention and/or treatment of pancreatic cancer, familial adenomatous polyposis (FAP), inflammatory bowel disease, durable bowel disease, and other pro-inflammatory diseases. In addition, research studies have suggested that many cancer preventive agents mediate their effects through inhibition of NF-kB and STAT-3 (Baek and Eling, 2006).

## ANTI-OXIDANTS AND CANCER THERAPY

The potential preventive anti-cancer effects of anti-oxidants found in high concentrations in many phytochemicals have become a major focus of research in recent years. Despite accumulating evidence to suggest an important role in cancer prevention, there has been much controversy over their potential therapeutic applications based on the fact that these agents block free radical formation as an important mediator of their anti-cancer effects (Stamatakos et al., 2006). Although free radicals may be important intracellular carcinogens, nevertheless, the formation of free radicals may mediate the cytotoxic tumor cell killing activities of many standard chemotherapy drugs as well as radiation (Block, 2004; D’Andrea, 2005). Among the barriers to conventional therapy presented by the tumor microenvironment, it is well-known that hypoxia inhibits effective radiation killing, due to its limiting effects on the production of ROS (Komarova and Wodarz, 2005). In addition, chemotherapy resistance associated with low oxygen concentrations affects the activity of drugs such as melphalen, bleomycin, and etoposide, all of which require molecular oxygen for their cell killing effects. Research on chemotherapy resistance further suggests that stem cells with low concentrations of ROS may be an important cause of treatment failure (Achuthan et al., 2011). Hypoxia can also induce cell cycle arrest and resistance to apoptosis, both of which can dramatically decrease the efficacy of drugs that target proliferating cells (Michor et al., 2005; Kozusko et al., 2007). That said, the purpose of this discussion is not to explore the potential use of anti-oxidants in the context of their potential inhibitory effects when used in conjunction with cytotoxic chemotherapy or radiation, but rather to explore other potentially important relationships between anti-oxidant activity and cancer as they relate to the development of incipient cancers and tumor progression.

To address this question, it is necessary to explore the biological basis of the potential anti-cancer therapeutic effects of anti-oxidants. Of particular importance is the question of whether anti-oxidants can prevent the formation of incipient tumors and/or affect parameters of tumor growth behavior involving ROS (Kennedy, 1987; Wardman, 2001; Brown and Wilson, 2004). Abnormal tumor vasculature is a primary cause of hypoxia, intratumoral acidic conditions and increased interstitial fluid pressure (IFP) of the tumor microenvironment (Boucher et al., 1990).

Moreover, based on their effects on mitochondrial function, it is entirely possible that the formation of oxygen free radicals may contribute to the development of tumor-associated hypoxia as a result of mitochondrial dysfunction. Normal PO2 oxygen ranges between 10 and 80 mmHg; many solid tumors contain PO2 at less than 5 mmHg. Elevated tumor acidity compared to normal tissues is associated with lactate accumulation due to the spacio-temporal pH gradients resulting from the metabolic state of tumor cells and the ion pumping mechanisms that vary significantly in tumors (Bonuccelli et al., 2010a; Pavlides et al., 2010a). The resulting activation of hypoxia inducible factor (HIF-1a) (Wang and Semenza, 1995) is associated with solid tumor progression and has been shown to drive many of the survival and metastatic pathways that are characteristic of advanced disease (Martinez-Outschoorn et al., 2010a,c).

Under normal physiological conditions, a small fraction of oxygen consumed by mitochondria is converted to superoxide anions, H_2_O_2_, hydroxyl radicals, and other ROS. Within specific concentration limits, ROS regulate cell functions, acting as a second messenger to activate transcription factors NF-kB and activator protein 1 (AP-1). Excess ROS production, however, is destructive. Overproduction of ROS by damaged mitochondria may activate inflammatory pathways linked to cancer (Lisanti et al., 2010; Martinez-Outschoorn et al., 2010c; Pavlides et al., 2010b; Toullec et al., 2010). Moreover, mitochondrial production of ROS as a byproduct of oxidative respiration is accelerated by the aging process. Impairment of the electron transport chain results in enhanced production of ROS in mitochondria due to incomplete reduction of oxygen (Pavlides et al., 2010b). Anti-oxidants may derail this process by blocking inflammatory responses to ROS as well as by preventing the accumulation of intracellular ROS. Anti-oxidant enzymes that block ROS are manganese glutathione reductase (GR), catalase (CAT), manganese +2-dependent superoxide dismutase (MnSOD), copper zinc superoxide dismutase (SODCu/Zn), and glutathione peroxidase (GPx; Bravard et al., 1992; Behrend et al., 2005; Oberley, 2005; St. Clair et al., 2005). Their activities decline with aging, resulting in age-dependent damage to DNA, RNA, lipids, and proteins. Moreover, bioenergetic functions decline with age. Oxidative damage to mitochondrial DNA is much more extensive than to nuclear DNA and is associated with increased glycolysis that may be blocked by glycolytic inhibitors resulting in what has been called a “reverse Warburg effect” (Pavlides et al., 2009; Bonuccelli et al., 2010b).

The overall result is elevated levels of oxidative stress. ROS may induce stress responses to maintain energy metabolism, but at high levels this causes broad-spectrum oxidative damage that may elicit apoptosis by inducing membrane permeability changes in mitochondria resulting in the release of cytochrome C. Oxidative damage also produces deletions and duplications in mitochondrial DNA, a process that increases with age in many human tissues, further contributing to mitochondrial dysfunction. In this context, mitochondria may be regarded as biosensors of oxidative stress in the cell (Martinez-Outschoorn et al., 2010b).

**FIGURE 8:**
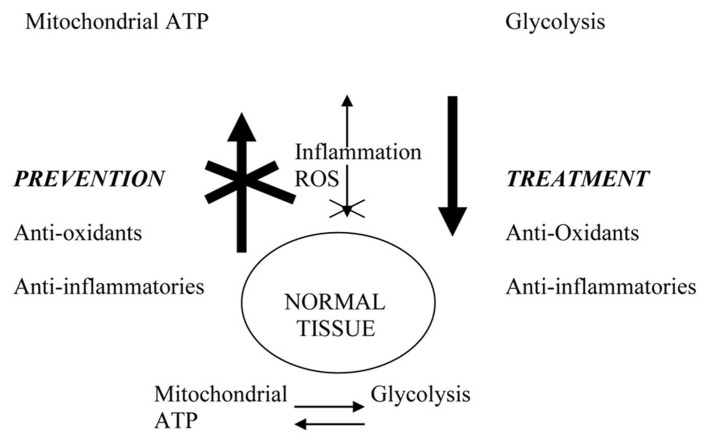
**Physiological connections that may link cancer prevention and treatment**. Excessive ROS (reactive oxygen species) due to aging or environmental exposure damage mitochondria and promote glycolytic switch characteristic of tumor cells. Inflammation and ROS activate NF-kB and other transcription factors that drive tumor progression. Anti-inflammatory agents and anti-oxidants prevent cancer by blocking systemic and microenvironmental changes that promote incipient tumor development and systemic disease progression. In the therapeutic setting, their long-term use restores systemic environment that blocks disease progression and/or recurrence.

Thus, there appears to be a cyclical interaction between mitochondrial respiration, which is an important source of ROS due to electron leakage from the respiratory chain, and the destructive effects of excessive ROS production on mitochondrial function (see **Figure 8**). Harman suggested that mitochondria are major targets of free radical attack that leads to aging, a concept embodied in the “free radical theory of aging.” Miquel and Bertoni-Freddari (2000) showed that oxidative damage to mitochondrial DNA and lipofuscin pigment formation concurrently increase in aging. Linnane et al. (1989) hypothesized that the accumulation of somatic mutations in mitochondrial DNA is a major contributor to aging and degenerative diseases, a concept embodied in the “mitochondrial theory of aging.” Moreover, Bonuccelli et al. (2010b) suggested that enhanced lactate and ketone production from elevated glycolysis associated with depressed mitochondrial oxidative phosphorylation may drive metastasis via oxidative metabolism.

The loss of stromal caveolin-1 (Cav-1) has been linked to a “reverse Warburg effect” (Warburg, 1956), resulting in autophagy and mitophagy in tumor-associated fibroblasts that provide additional energy-producing intermediates to tumor cells in the microenvironment that support tumor growth (Pavlides et al., 2009; Lisanti et al., 2010; Pavlides et al., 2010a,b,c) Moreover, low levels of stromal Cav-1 is a biomarker for poor prognosis in some common cancers, such as prostate cancer and breast cancer (Mercier et al., 2008; Di Vizio et al., 2009; Witkiewicz et al., 2009a). In patients with triple negative breast (TNB) cancer, high levels of stromal Cav-1 correlate with survival rates of over 75% in 12 years. In contrast, TNB patients with very low levels of stromal Cav-1 have a 5-year survival rate of less than 10% (Finak et al., 2008; Sloan et al., 2009; Witkiewicz et al., 2009b, 2010). In the context of this discussion, it should be noted that anti-oxidants such as *N*-acetyl-cysteine, quercetin, and metformin as well as chloroquine, which inhibits autophagy, have been shown to prevent loss of stromal Cav-1 in tissue co-culture systems (Trimmer et al., 2011).

## PREVENTION VERSUS TREATMENT: POTENTIAL PHYSIOLOGICAL CONNECTIONS

There is increasing evidence that anti-oxidant activity may play a role in suppressing the tumor phenotype. Suppression of the glioma phenotype by Mn^+^^+^SOD overexpression has been reported (Zhong et al., 1997) as well as in SV40 transformed lung fibroblasts (Yan et al., 1996; Oberley, 2001). Additional research suggests that up-regulation of Mn^+^^+^SOD may be an important target for anti-cancer therapeutics (Duan et al., 2003; Pani et al., 2004; Venkataraman et al., 2005). With respect to anti-oxidant effects on ROS-associated aging processes, research has shown that oral administration of anti-oxidants protects rats and mice from glutathione oxidation and mitochondrial damage. Additional research findings that strongly suggest that oxidative damage results in mitochondrial defects in respiration and oxidative phosphorylation (Kovacic and Osuna, 2000; Wardman, 2001). Clearly, more studies are needed in this area; however, these research studies suggest that an important component of anti-oxidant preventive may involve the prevention of ROS-induced mitochondrial damage, which contributes importantly not only to aging, but also to cancer, perhaps by activating the glycolytic switch that characterizes cancer cell metabolism.

One question that needs to be addressed in the context of developing a new therapeutic paradigm is whether agents that prevent cancer by blocking inflammation may also act therapeutically by preventing cancer spread and recurrence. Do agents that block inflammation, such as aspirin, act only at the level of tumor initiation by inhibiting inflammatory pathways that may contribute to the development of an incipient pre-malignant state? Or, is there evidence that their activities go beyond this to affect pre-established malignant tumors within the body to block their further progression? In other words, do disease progression and the development of systemic disease require inflammatory processes for their sustenance? The answer to these questions is a probable “yes,” based on clinical data results (see previous section), the observed cytotoxic effects of aspirin in cultured tumor cells, and physiological assessments of the presence of elevated levels of several biomarkers for inflammation in patients with many types of advanced cancer. It is entirely possible that some of the apparent chemopreventive properties of anti-inflammatory agents may be operating at the level of pre-established incipient malignancies in which further tumor progression and the onset of overt disease are significantly delayed or do not occur at all. It is important to carry out studies to establish this possibility as a rationale for the use of this approach in long-term, preventive modalities. If so, then anti-inflammatory agents might be developed for use in long-term maintenance therapeutic approaches in the management of malignant disease as well as in cancer prevention.

The inclusion of long-term maintenance therapy involving the use of any anti-inflammatories must be evaluated in this context. The concept of long-term maintenance therapy approaches to prevent disease recurrence is further supported by longitudinal studies on the long-term use of tamoxifen and aromatase inhibitors in the management of breast cancer (Early Breast Cancer Trialists’ Collaborative Group et al., 2011). The consensus of these clinical studies is that, despite the occurrence of side effects, these approaches may be life-saving in terms of preventing disease recurrence by targeting growth factor and hormonally activated pathways important to sustaining tumor progression and abnormal growth pathways contributing to disease recurrence.

Another point that should be emphasized involves the fact that successful therapy with gene-targeted drugs such as Gleevec, tamoxifen, and aromatase inhibitors requires ongoing treatment involving a continuous dosing regimen that follows a very different protocol from that generally followed in cytotoxic chemotherapy. In the case of gene-targeted drugs such as Gleevec, the patient will receive the drug on a long-term basis as a form of maintenance therapy to prevent the establishment of drug-resistant clones. The need for continuous maintenance therapy in the successful management of patients with chronic myelogenous leukemia (CML) using Gleevec suggests that successful cancer management in general may require long-term maintenance therapy in order to achieve stable remissions and to prevent the development of acquired drug resistance. These clinical successes in long-term cancer management underscore the importance of developing therapeutic approaches that can be used in long-term maintenance therapy to prevent disease recurrence.

This approach, of course, is not possible using conventional chemotherapeutics; due to their high-level toxicity, it would be impossible to use cytotoxic drugs for long-term maintenance therapy regimens. This, therefore, represents a basic therapeutic modality that cannot be entertained in the context of conventional chemotherapy drugs. Nevertheless, the successful management of many types of cancer may require an approach that involves some type of long-term maintenance therapy in order to prevent disease recurrence. An important therapeutic goal for future research, therefore, may involve the development of therapeutics designed for use in long-term maintenance therapy. These agents, though specific in their cell-targeted inhibitory effects, nevertheless impact essential pathways that drive the malignant phenotype. Perhaps nothing less will do.

The concepts of anti-inflammatory and anti-oxidant approaches to cancer therapy involve a very basic hypothesis, that similar mechanisms may be associated with their cancer preventive effects and therapeutic effects. In other words, prevention and treatment may not be two different entities but rather may be multifaceted components necessary to create a systemic environment that prevents both the development and spread of malignancies within the body. By altering the tumor-promoting environment, either in pre-malignant, active or post-malignant conditions, these so-called preventive agents may help to maintain a system equilibrium that is resistant both to tumor development and disease recurrence. The paradigm that prevention and treatment are interconnected therapeutic approaches is based on the proposed interrelationship between physiological conditions associated with the development of incipient cancer and the progression to systemic disease. This paradigm suggests that the treatment of cancer is not simply regarded as a short-term effort to destroy tumor cells in patients with diagnosed disease but rather as both a preventive approach in healthy individuals to protect against the development of incipient cancers and also as a therapeutic approach in diagnosed patients to modify the system in such a way that the host no longer serves to support tumor growth within the body.

## CONCLUSION

For the past several decades, the prevailing therapeutic paradigm for the treatment of both disseminated leukemias/lymphomas as well as almost all solid tumor malignancies has involved the use of dose-dense, combined MTD cytotoxic chemotherapy. Current therapeutic protocols for the treatment of most cancers were developed initially in the context of treating childhood leukemias and lymphomas; their extended clinical applications to the treatment of solid tumor malignancies were based largely on the therapeutic successes observed, for example, in the treatment of HD and acute lymphoblastic childhood leukemia (ALL).

Despite these broad-spectrum applications to almost all currently used chemotherapy regimens, in only a few types of cancer do we see curative responses such as have been consistently observed in the treatment of childhood ALL and HD. Thus, the results of over a half-century of clinical trials have shown that the therapeutic approach of combined/dose-dense chemotherapy has not been successful in achieving its primary purpose, which is the induction of long-term disease-free survival in the majority of patients with systemic disease.

The clinical use of most conventional chemotherapy drugs involves the basic assumption that the cancer phenotype is characterized by continuous dysregulated cell proliferation. Therapeutic modeling in solid tumors was largely based on the growth parameters defined by the growth of leukemia cells; however, the vast differences in growth properties that distinguish solid tumor growth from that of leukemias and some lymphomas made this translational approach inappropriate. Research on tumor biology suggests that many solid tumors of diverse tissue types are heterogeneous collections of cells that are not consistently or uniformly proliferating at any given time. Fundamental differences between the growth properties of leukemias/lymphomas and solid tumors may thus explain key differences in chemotherapy sensitivities.

The association between chronic inflammation and ROS activity in the genesis of cancer is supported by clinical research studies that provide evidence for the preventive aspects of the long-term use of aspirin and anti-oxidants in the development of some common malignancies. Moreover, based on their effects on mitochondrial function, it is entirely possible that inflammation/ROS may contribute to abnormal tumor physiology that promotes tumor progression and metastasis.

The concept of long-term anti-inflammatory/anti-oxidant maintenance approaches to cancer therapy involves a very basic hypothesis, that similar physiological mechanisms may be responsible for both cancer preventive and therapeutic effects. Prevention and treatment may not be two different entities but rather may be multifaceted components necessary to create a systemic environment that prevents both the development and spread of malignancies within the body. By altering the tumor-promoting environment, either in pre-malignant, active or post-malignant conditions, these so-called preventive agents may help to maintain a system equilibrium that is resistant both to tumor development and disease recurrence.

## Conflict of Interest Statement

The author declares that the research was conducted in the absence of any commercial or financial relationships that could be construed as a potential conflict of interest.
